# Increased incidence of kidney diseases in general practice after a nationwide albuminuria self-test program

**DOI:** 10.1186/1471-2296-12-81

**Published:** 2011-08-03

**Authors:** Julia de Borst, Markus MJ Nielen, Robert A Verheij, François G Schellevis

**Affiliations:** 1 Faculty of Earth and Life sciences, VU University, Amsterdam, the Netherlands; 2 NIVEL (Netherlands Institute for Health Services Research), Utrecht, the Netherlands; 3 Department of General Practice/EMGO Institute, VU University Medical Center, Amsterdam, The Netherlands

## Abstract

**Background:**

To study the influence of a nationwide albuminuria self-test program on the number of GP contacts for urinary complaints and/or kidney diseases and the number of newly diagnosed patients with kidney diseases by the GP.

**Methods:**

Data were used from the Netherlands Information Network of General Practice (LINH), including a representative sample of general practices with a dynamic population of approximately 300.000 listed patients. Morbidity data were retrieved from electronic medical records, kept in a representative sample of general practices. The incidence of kidney diseases and urinary complaints before and after the albuminuria self-test program was compared with logistic regression analyses.

**Results:**

Data were used from 139 general practices, including 444,220 registered patients. The number of GP consultations for kidney diseases and urinary complaints was increased in the year after the albuminuria self-test program and particularly shortly after the start of the program. Compared with the period before the self-test program, more patients have been diagnosed by the GP with symptoms/complaints of kidney disease and urinary diseases (OR = 1.7 (CI 1.4 - 2.0) and OR = 2.1 (CI 1.9 - 2.3), respectively). The odds on an abnormal urine-test in the period after the self-test program was three times higher than the year before (OR = 3.0 (CI 2.4 - 3.6)). The effect of the self-test program on newly diagnosed patients with an abnormal urine test was modified by both the presence of the risk factors hypertension and diabetes mellitus. For this diagnosis the highest OR was found in patients without both conditions (OR = 4.2 (CI 3.3 - 5.4)).

**Conclusions:**

A nationwide albuminuria self-test program resulted in an increasing number of newly diagnosed kidney complaints and diseases the year after the program. The highest risks were found in patients without risk factors for kidney diseases.

## Background

Because of the increasing prevalence rate of lifestyle-related diseases such as cardiovascular diseases and diabetes mellitus [1-3], but also chronic kidney diseases, population-based prevention strategies are needed to prevent future problems for patients and to reduce costs for health care. Therefore, screening for risk factors of these chronic diseases in the general population becomes more popular. In 2006, the Dutch Kidney Foundation started a program to detect persons at risk for chronic renal failure (CRF) in the Netherlands by offering a free self-test for albuminuria [4]. Patients with CRF have structural abnormalities of the kidney resulting in decreased kidney function, necessitating dialysis or kidney transplantation [5].

With advertisements on the radio, television, newspapers and the Internet, Dutch adults over the age of 18 were invited to order the free albuminuria self-test via the Internet or by telephone in the period September until November 2006. The presence of an increased amount of albumin in the urine is an indicator for worsening kidney function and increases the risk of developing CRF. In case of a positive test result, participants were advised to visit their General Practitioner (GP) for additional examination and treatment [4]. In the Netherlands, the GP has a gatekeeper role for access to specialized care. All Dutch citizens are listed with a general practice and the GP is usually the first professional to be consulted with health problems.

In a previous study the usefulness of the albuminuria self-test program was investigated in a population of participants who ordered the self-test [4,6]. From that study it was concluded that more than half of the adult Dutch population was aware of the self-test program and over one million persons (about 9% of the Dutch adult population) ordered the self-test via the Internet in the first 30 days of the program. Also GP visits following a positive test result have contributed to newly detected patients with kidney diseases, hypertension and/or diabetes mellitus. Besides these positive findings, there were also some negative side-effects, which are common in screening programs in the general population. A large number of (probably) false positive test results were found. This causes worries among the test users, unnecessary use of health care and therefore unnecessary costs. Also, due to the low a-priori risk on CRF in the general population, it would have been better to test in populations at high risk. Finally, only a small proportion of the persons with a positive test result (25%) visited a GP for additional examination and/or treatment within eight weeks after testing [4].

Given the high awareness of the self-test program, it is likely that the program not only resulted directly in newly detected diseases, but also indirectly led to an increased number of newly detected kidney diseases, since 1) instead of self-testing, patients could have decided to visit their GP for testing the renal function, and 2) GPs could have become more focused on the detection of kidney diseases as a result of the program. In our previous study questionnaires of participants were used and therefore it was not possible to determine the consequences of the self-test program for the GP.

Therefore, in this study we have investigated the influence of the screening program on the number of GP contacts for urinary complaints and/or kidney diseases and the number of newly diagnosed patients with kidney diseases by the GP. Accordingly, the following research questions were formulated: 1) Are there differences in the total number of GP contacts for urinary complaints and/or kidney diseases before and after the albuminuria self-test program?, 2) Are there differences in the number of newly diagnosed patients with kidney diseases by the GP before and after the albuminuria self-test program?, and 3) Are there differences in the number of newly diagnosed patients in persons with hypertension and/or diabetes mellitus and patients without these risk factors before and after the albuminuria self-test program?

## Methods

### Netherlands Information Network of General Practice

Data were used from the Netherlands Information Network of General Practice (LINH). Data were retrieved from electronic medical records, kept in a representative sample of general practices with approximately 300.000 listed patients in 2008 [7]. Data include information on consultations, morbidity, prescriptions and referrals. The characteristics of the study population (practices as well as patients) were comparable with the Dutch population in terms of age and gender [8]. Diagnoses were recorded using the ICPC-1 coding system (International Classification of Primary Care) [9]. When issuing a prescription, a diagnostic code was recorded, and the selected drug was automatically linked to the Anatomical Therapeutic Chemical (ATC) Classification System http://www.whocc.no/.

The study was carried out according to Dutch legislation on privacy. The privacy regulation of the study was registered at the Dutch Data Protection Authority. According to Dutch legislation, nor obtaining informed consent nor approval by a medical ethics committee was obligatory for observational studies.

### Design and study population

In this study the number of contacts with the GP for kidney diseases and/or urinary complaints and the number of newly diagnosed patients with kidney diseases were compared between the year before and after the albuminuria self-test program. The period before the program was from 1 September 2005 to 31 August 2006 (Period 1) and the period after the program was from 1 November 2006 to 31 October 2007 (Period 2). The two months in between were not included in the study, because there was a delay between the start of the program at 1 September 2006 and the first GP visits by participants with a positive test result (caused by delivering of the self-test by post, testing for albuminuria and eventually visiting the GP).

Data are used from a dynamic population and include all consultations from patients who were registered at a LINH practice at any time during the study period (1 September 2005 to 31 October 2007). We used a dynamic population, because the group of registered patients at a GP practices is not fixed (patients change their GP, relocate or die). In case a patient was not registered the whole study period -when a general practice did not deliver data during the whole study period or when patients were registered temporarily-, a 'time at risk' was calculated. Patients who were under the age of 18 at the start of the self-test program (1 September 2006) were excluded, because the self-test was meant to be used by adults.

Per selected patient we determined if they were diagnosed in a period of three years before the first study year with one of the following kidney diseases/urinary complaints: U07 (Urine symptom/complaint, other), U14 (Kidney symptom/complaint), U27 (Fear of urinary disease), U29 (Urinary symptom/complaint, other), U98 (Abnormal urine test) and U99 (Urinary disease, other (renal failure)). Furthermore the presence of risk factors for chronic kidney disease, i.e. diabetes mellitus (ICPC code T90), hypertension (ICPC code K86 and/or K87), at the start of the study period was determined.

### Statistical analyses

Three different statistical analyses were performed. First, a descriptive analysis was performed to determine the course of the number of consultations per week with the GP for kidney diseases and urinary complaints (one of the following ICPC codes: U07, U14, U27, U29, U98 or U99) over the whole study period. The number of consultations was divided by the total number of contacts in that week to correct for seasonal fluctuations in the number of GP contacts. To adjust for outliers, a 4-week moving average was calculated and plotted.

Second, per kidney disease/urinary complaint the absolute number of newly diagnosed patients was determined for the period before and after the self-test program. In addition, the absolute number of cases and the total 'time at risk' of all patients in the specified periods were used to calculate person-time incidence rates (IR) per 10,000 person years. The IR of both periods were used to calculate incidence rate ratio's (IRR) with 95% confidence intervals. IRs and IRRs were calculated for the total study population and stratified by age (age groups 18-45 years, 46-65 years and 66 years and older), presence of hypertension and presence of diabetes mellitus, which are all risk factors for chronics kidney diseases.

Third, per kidney disease/urinary complaint the number of newly diagnosed patients by the GP in the period before the self-test program were compared with the number of newly diagnosed patients in the period after the self-test program with logistic regression analyses. The models were adjusted for age and gender and the presence of hypertension and diabetes mellitus. Odds ratios (ORs) are reported with 95% confidence intervals. In addition, it was tested if the presence of these risk factors for kidney diseases modified the effect of the self-test program on the number of newly detected kidney diseases by adding interaction terms to the model. If the interaction was statistically significant (p-value < 0.05), ORs were presented for subgroups. All statistical analyses were performed with Stata 11.

## Results

### Characteristics of the study population

Data from 81 out of the 85 (95.3%) participating LINH practices in 2005 could be used for statistical analyses. Four practices had to be excluded because of missing data. In 2006 and 2007, 58 new general practices were included in LINH. In total, data were used from 139 general practices, including 444,220 registered patients. Of these patients, 227,393 were female (51%) and the largest age group was 36-45 years old (see table 1). At the start of the study period, 6,005 patients were already diagnosed with a kidney disease or an urinary complaint, 34,022 patients were diagnosed with hypertension and 12,552 patients with diabetes mellitus.

**Table 1:** Population characteristics at baseline (1 September 2005)

	Number of cases (*n*)	Percentage (%)
**Total**	444,220	
		
**Gender**		
Female	227,393	51.2
Male	216,827	48.8
		
**Age groups**		
18-25 years	66,677	15.0
26-35 years	83,919	18.9
36-45 years	91,255	20.5
46-55 years	76,679	17.3
56-65 years	58,968	13.3
66-75 years	37,783	8.5
76-85 years	22,997	5.2
85+ years	5,942	1.3

### Number of GP contacts for kidney diseases

**Figure 1:**
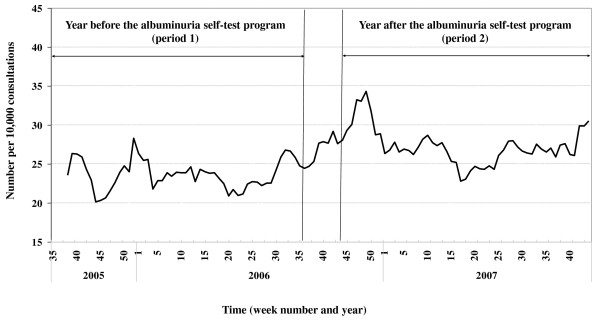
**Number of GP consultations per week for kidney diseases/urinary complaints per 10,000 consultations**.

The number of GP consultations for kidney diseases and urinary complaints per 10,000 consultations is plotted in Figure 1. The number of GP consultations fluctuated between 20 and 25 per 10,000 consultations in period 1. This rate increased by approximately 5 per 10,000 consultations to 25 to 30 per 10,000 consultations in period 2. Moreover, the number of consultations increased in the first four weeks of period 2 to as high as an additional 10 per 10,000 consultations.

### Newly diagnosed kidney diseases in the period before and after the self-test program

The IRs of kidney diseases/urinary complaints in the period before and after the albuminuria self-test program are shown in table 2. These IRs were used to calculate IRRs for the total population and subgroups. In the total study population, statistical significant IRRs were found for the diagnoses U14 (Kidney symptom/complaint), U98 (Abnormal urine test) and U99 (Urinary disease, other (renal failure)). For the diagnosis U14 the IR increased from 4.4 to 6.9 per 10,000 person years, resulting in a 1.6 times higher IR in the period after the self-test program compared with the year before the program. This effect was found in all age groups and in subjects without hypertension and diabetes mellitus. The IR of the diagnoses U98 and U99 increased from 3.6 to 10.0 per 10,000 person years and from 13.8 to 25.8 per 10,000 person years, respectively. This resulted in IRRs of 2.8 (CI 2.3 - 3.4) and 1.9 (CI 1.7 - 2.1) for the diagnoses U98 and U99, respectively. The increase of the number of abnormal urine tests (U98) was the highest in the age category 18-45 years old (IRR 4.2 (CI 2.8 - 6.3)). The IR of urinary diseases (U99) was increased in all subgroups the year after the program and the IRR varied between 1.7 and 2.1.

**Table 2:** Incidence rates of kidney diseases/urinary complaints in general practice before (period 1) and after (period 2) the albuminuria self-test program per 10,000 person years in the total population and in subgroups.

	Period 1		Period 2		Incidence Rate Ratio^2 ^(95% CI)
	
	Absolute numbers	Person-time incidence rate^1^	Absolute numbers	Person-time incidence rate^1^	
***ICPC U07: Urine symptoms/complaint***	***548***	***14.9***	***625***	***15.6***	***1.0 (0.9 - 1.2)***
18-45 years old	220	11.1	257	11.8	1.1 (0.9 - 1.3)
46-65 years old	146	12.9	186	15.2	1.2 (0.9 - 1.5)
> 65 years old	182	32.9	182	30.6	0.9 (0.8 - 1.1)
Hypertension, yes	96	33.5	86	33.2	1.0 (0.7 - 1.3)
Hypertension, no	452	13.3	539	14.4	1.1 (1.0 - 1.2)
Diabetes mellitus, yes	61	52.4	49	46.2	0.9 (0.6 - 1.3)
Diabetes mellitus, no	487	13.7	576	14.8	1.1 (1.0 - 1.2)
					
***ICPC U14: Kidney symptoms/complaint***	***161***	***4.4***	***277***	***6.9***	1.6 (1.3 - 1.9)*
18-45 years old	63	3.2	117	5.4	1.7 (1.2 - 2.3)*
46-65 years old	61	5.4	92	7.5	1.4 (1.0 - 2.0)*
> 65 years old	37	6.7	68	11.3	1.7 (1.1 - 2.6)*
Hypertension, yes	29	10.1	30	11.5	1.1 (0.7 - 2.0)
Hypertension, no	132	3.9	247	6.6	1.7 (1.4 - 2.1)*
Diabetes mellitus, yes	14	11.9	12	11.1	0.9 (0.4 - 2.2)
Diabetes mellitus, no	147	4.1	265	6.8	1.6 (1.3 - 2.0)*
					
***ICPC U27: Fears of urinary disease***	***424***	***11.6***	***450***	***11.3***	***1.0 (0.9 - 1.1)***
18-45 years old	207	10.5	204	9.4	0.9 (0.7 - 1.1)
46-65 years old	123	10.9	137	11.2	1.0 (0.8 - 1.3)
> 65 years old	94	17.0	109	18.3	1.1 (0.8 - 1.4)
Hypertension, yes	87	30.7	51	19.9	0.6 (0.4 - 0.9)*
Hypertension, no	337	10.0	399	10.7	1.1 (0.9 - 1.2)
Diabetes mellitus, yes	37	32.0	17	16.1	0.5 (0.3 - 0.9)*
Diabetes mellitus, no	387	10.9	433	11.2	1.0 (0.9 - 1.2)
					
***ICPC U29: Urinary symptoms/complaint***	***94***	***2.6***	***112***	***2.8***	***1.1 (0.8 - 1.5)***
18-45 years old	32	1.6	47	2.2	1.3 (0.8 - 2.2)
46-65 years old	27	2.4	28	2.3	1.0 (0.5 - 1.7)
> 65 years old	35	6.3	37	6.2	1.0 (0.6 - 1.6)
Hypertension, yes	14	4.8	16	6.1	1.3 (0.6 - 2.8)
Hypertension, no	80	2.4	96	2.6	1.1 (0.8 - 1.5)
Diabetes mellitus, yes	6	5.1	6	5.6	1.1 (0.3 - 4.1)
Diabetes mellitus, no	88	2.5	106	2.7	1.1 (0.8 - 1.5)
					
***ICPC U98: Abnormal urine test***	***132***	***3.6***	***400***	***10.0***	2.8 (2.3 - 3.4)*
18-45 years old	32	1.6	146	6.7	4.2 (2.8 - 6.3)*
46-65 years old	53	4.7	142	11.6	2.5 (1.8 - 3.5)*
> 65 years old	47	8.4	112	18.7	2.2 (1.6 - 3.2)*
Hypertension, yes	38	13.2	31	11.9	0.9 (0.5 - 1.5)
Hypertension, no	94	2.8	369	9.9	3.6 (2.8 - 4.5)*
Diabetes mellitus, yes	26	22.3	27	25.3	1.1 (0.6 - 2.0)
Diabetes mellitus, no	106	3.0	373	9.6	3.2 (2.6 - 4.0)*
					
***ICPC U99: Urinary disease***	***507***	***13.8***	***1031***	***25.8***	1.9 (1.7 - 2.1)*
18-45 years old	51	2.6	120	5.5	2.1 (1.5 - 3.0)*
46-65 years old	118	10.4	247	20.2	1.9 (1.6 - 2.4)*
> 65 years old	338	61.1	664	112.5	1.8 (1.6 - 2.1)*
Hypertension, yes	193	67.7	293	114.4	1.7 (1.4 - 2.0)*
Hypertension, no	314	9.3	738	19.7	2.1 (1.9 - 2.4)*
Diabetes mellitus, yes	113	97.7	139	132.7	1.4 (1.1 - 1.8)*
Diabetes mellitus, no	394	11.1	892	22.9	2.1 (1.8 - 2.3)*

^1^The absolute number of cases divided by the 'time at risk' of all patients in specified period (per 10,000 person years)

^2 ^The incidence rate ratio is the person-time incidence rate of period 2 divided by the person-time incidence rate of period 1

* P < 0.05

### Logistic regression analyses

The results of the logistic regression analyses are shown in table 3. Odds ratios (ORs), adjusted for age, gender and the presence of hypertension and diabetes mellitus, were calculated for six kidney diseases/urinary complaints. There were no significant ORs found for the diagnoses U29 (Urinary symptom/complaint, other) and U27 (Fear of urinary disease). However, for diagnosis U27 we found statistical significant interaction with the presence of hypertension and diabetes mellitus (p = 0.01 and p = 0.02, respectively). Patients with hypertension and diabetes mellitus had a lower odds of being diagnosed with fear of urinary disease (U27) the period after the self-test program (OR = 0.6 and OR = 0.5, respectively).

**Table 3:** Risk of being newly diagnosed with a kidney disease in general practice after the albuminuria self-test program compared with the period before the program^#^

	Hazard Ratio (95% CI)	P-value
ICPC U07: Urine symptoms/complaint	1.1 (1.0 - 1.3)	0.05
U07 * Diabetes mellitus		0.31
U07 * Hypertension		0.62
		
ICPC U14: Kidney symptoms/complaint	1.7 (1.4 - 2.0)	< 0.001
U14 * Diabetes mellitus		0.17
U14 * Hypertension		0.17
		
ICPC U27: Fears of urinary disease	1.0 (0.9 - 1.2)	0.53
U27 * Diabetes mellitus		0.02
U27 * Hypertension		0.01
		
ICPC U29: Urinary symptoms/complaint	1.2 (0.9 - 1.5)	0.28
U29 * Diabetes mellitus		0.99
U29 * Hypertension		0.69
		
ICPC U98: Abnormal urine test	3.0 (2.4 - 3.6)	< 0.001
U98 * Diabetes mellitus		< 0.001
U98 * Hypertension		< 0.001
		
ICPC U99: Urinary disease	2.1 (1.9 - 2.3)	< 0.001
U99 * Diabetes mellitus		< 0.01
U99 * Hypertension		0.06

^#^adjusted for gender, age, hypertension and diabetes

The year after the self-test program, there was a higher odds of being diagnosed with the following diagnoses: U07 (Urine symptom/complaint, other), U14 (Kidney symptom/complaint), U98 (Abnormal urine test) and U99 (Urinary disease, other (renal failure)) with the highest OR for an abnormal urine test (OR = 3.0 (CI 2.4 - 3.6)). The effect of the self-test program on newly diagnosed patients with an abnormal urine test (ICPC code U98) was modified by both the presence of hypertension and diabetes mellitus. For this diagnosis, the highest OR was found for patients without both conditions (OR = 4.2 (CI 3.3 - 5.4)). Furthermore, for the diagnosis 'Urinary disease' (ICPC code U99), the odds of being newly diagnosed was higher in patients without diabetes mellitus (OR = 2.3 (CI 2.0-2.6) versus OR 1.4 (CI 1.1-1.9) in patients with diabetes).

## Discussion

The number of GP consultations for kidney diseases and urinary complaints increased in the year after the albuminuria self-test program and particularly shortly after the start of the program. Compared with the year before the self-test program, 1.7 times more patients have been diagnosed by the GP with symptoms/complaints of kidney disease and 2.1 times more patients with urinary diseases. The odds of an abnormal urine-test in the period after the self-test program was 3.0 times higher than the year before. The effect of the self-test program on newly diagnosed patients with an abnormal urine test was modified by both the presence of the risk factors hypertension and diabetes mellitus. For this diagnosis the highest OR was found in patients without both conditions (OR = 4.2).

In a previous study, the effects on participants of the albuminuria self-test program were evaluated using a questionnaire eight weeks after the start of the self-test program [4,6]. There were positive results (high awareness and newly detected diseases), but also some negative side-effects, such as false positive test results and a low number of persons who visited their GP after a positive test result [4]. With the results of that study it was not possible to determine the consequences of the self-test program for the GP. Therefore, in the present study data from electronic medical records of GPs were used to investigate the influence of the nationwide self-test program in a large group of patients representative for the Dutch population. In addition to this previous study also the indirect influences of the program on the number of newly detected kidney diseases could be measured over the whole year after the self-test program.

Previous studies have shown positive effects of mass media campaigns on the effectiveness of screening. Awareness of a mass media campaign is not only associated with higher awareness of symptoms of a disease, but also increases the screening rate [10-12]. Two studies reported that the response on cervical cancer screening increased by 18-27% during a mass media campaign [10,11]. This is in line with the results found in this study. Despite the positive effects of mass media campaigns on screening, there are only short-term effects [10,11]. Mass media campaigns should be combined with other approaches to achieve long-term effects [10,12].

In this study we estimated the impact of a nationwide self-test program on the number of newly diagnosed patients with kidney diseases in general practice. Although we used data from a large representative network of GPs, this study has also a few limitations. For instance, we could not correct for all potential confounding factors in the statistical analyses. Socio-economic status, ethnic background, blood glucose levels, high-density lipoprotein (HDL) levels and cigarette smoking are not recorded by GPs in a systematic way and therefore could not be used in this study [13,14]. Furthermore, the influence of the self-test program on the number of newly diagnosed kidney complaints and disease could be underestimated because of the used registration method of the participating GPs. It is likely that a part of the less specific kidney complaints have been coded with an ICPC code in ICPC chapter A 'General and unspecified'. On the other hand, the attention for early detection of kidney diseases has increased the last several years, which could have partly explained the increased incidence ratio's. Finally, the increased incidence rates in the year after the self-test program could be caused by early diagnosis of asymptomatic patients and/or patients that would never be diagnosed without screening, because of death before symptom onset or a low risk of progression to end stage renal disease. It is unknown to what extent these factors have biased our results.

We found that the risk of being newly diagnosed by the GP with kidney diseases and/or urinary complaints one year after a nationwide albuminuria self test program was higher, especially for patients without risk factors for kidney diseases (diabetes mellitus and hypertension). The latter could be a result of periodic screening on kidney diseases of patients with diabetes and hypertension by the GP. According to the guidelines of the Dutch College of General Practitioners, patients with hypertension and diabetes should be monitored every year by the GP [15-17].

## Conclusions

It can be concluded that a nationwide albuminuria self-test program resulted in an increasing number of newly diagnosed kidney complaints and diseases the year after the program. The highest numbers were found in patients without risk factors for kidney diseases.

## Competing interests

The authors declare that they have no competing interests.

## Authors' contributions

JDB and MMJN performed the study according to protocol, analyzed the data and wrote the manuscript. RAV and FGS initiated and supervised the study and developed the study protocol. All authors read and approved the final manuscript.

## Pre-publication history

The pre-publication history for this paper can be accessed here:

http://www.biomedcentral.com/1471-2296/12/81/prepub
